# Exploring Challenges and Co‐Developing a Nutritional Resource for Antenatal Care: A Participatory Stakeholder Engagement Study With Midwives and Public Involvement

**DOI:** 10.1111/hex.70633

**Published:** 2026-03-02

**Authors:** Julie Abayomi, Helen Richards, Camila Benavides, Ekaterina Reyneke, Lisa Newson

**Affiliations:** ^1^ Health Research Institute Edge Hill University Ormskirk UK; ^2^ Liverpool Women's Hospital NHS Trust Liverpool UK; ^3^ School of Psychology Liverpool John Moores University Liverpool UK

**Keywords:** midwives, nutrition, pregnancy, weight

## Abstract

**Background:**

It is important that midwives discuss good nutrition and optimal weight during antenatal appointments, yet this rarely happens. Earlier research suggests that limited time, plus insufficient knowledge and skills are barriers to this.

**Aim:**

To engage antenatal midwives in stakeholder discussions to explore their perspectives on the design and delivery of a nutrition resource.

**Methods:**

This qualitative study used a Participatory Action Research (PAR) approach. Twenty‐six midwives (19 community‐based; 7 hospital‐based) were purposely recruited from four NHS sites in Northwest England. Four stakeholder workshops were co‐designed and facilitated by researchers. In Phase 1, midwives reflected on current practice and challenges in delivering diet and weight advice. In Phase 2, they co‐developed ideas for practical, acceptable nutrition resources. Data collection included participant‐generated artefacts, post‐it notes, visual maps, field notes, and verbatim reflections. Data were analysed inductively using Reflexive Thematic Analysis, and reflexivity was maintained throughout, recognising researchers' influence within this participatory design.

**Results:**

Two master themes were developed: (1) “We Want to Help, But We're Not Trained for This”, highlighting systemic, professional, and emotional barriers to providing support and (2) “Make It Real and Make It Work”, midwives' co‐produced recommendations for inclusive tools and training that are realistic, culturally sensitive, and integrated into existing workflows.

**Discussion and Conclusion:**

Midwives expressed a clear need for improved education and resource support. Despite systemic constraints, they co‐produced practical and implementable solutions. Supporting midwives through evidence‐based, context‐specific tools and training may enhance nutrition conversations in antenatal care and improve maternal and infant health outcomes.

**Patient or Public Contribution:**

Three culturally diverse Patient and Public Involvement (PPI) representatives were recruited to assist with the validation of the analytical findings. One was a midwife working in antenatal care; the second was a recent service user (a postnatal woman) with an Eastern European background, and the third was a midwife (currently on maternity leave) with a South American background. Commentary from these PPI representatives was used to validate the analysis and support the interpretation of the data. Additionally, they were invited to provide commentary on the draft manuscript and have been included as co‐authors.

## 1 Background

Good nutrition and optimal weight gain during pregnancy are essential for positive pregnancy outcomes. The World Health Organisation [1] highlights health professionals' crucial role in effectively communicating nutritional messages to pregnant women. The recently published UK Maternal and Child Nutrition clinical guidelines [2] provide more detailed recommendations than ever before, outlining the key discussions that healthcare professionals should incorporate into antenatal care. Compared to the previous guidelines [3], which were significantly less comprehensive, the updated guidance emphasises a broader range of essential topics.

These include the benefits of healthy eating and drinking habits, not only for the pregnant individual but also for their baby and the wider family. Healthcare professionals are encouraged to offer advice on foods and drinks that should be promoted or avoided, ensuring that recommendations align with acceptable and available options. Addressing common myths about dietary intake during pregnancy is also highlighted, helping to dispel misconceptions about what and how much to eat. Common myths that midwives encounter include beliefs such as ‘eating for two’ during pregnancy, the idea that all forms of fish should be avoided, or that cravings indicate nutritional deficiencies. These misconceptions can lead to confusion or harmful dietary choices if not adequately addressed. Prior research by Abayomi et al. [4] highlighted that women frequently receive inconsistent or contradictory information during pregnancy, often shaped by social media or familial advice rather than evidence‐based guidance. Additionally, the guidelines reinforce the importance of appropriate supplement use, particularly folic acid and vitamin D, as well as the role of physical activity in maintaining maternal well‐being. Sensitive communication about weight is also a key consideration, with an emphasis on avoiding stigma while supporting healthy weight management throughout pregnancy [2]. By providing a more structured and evidence‐based framework, these updated guidelines encourage midwives and other healthcare professionals to deliver clearer, more effective nutritional support, ultimately contributing to improved maternal and child health outcomes.

Research highlights that pregnant and postnatal women value healthy eating advice as a key component of antenatal care; however, they frequently report that the information they receive is inconsistent, superficial, and often focused primarily on foods to avoid rather than offering practical, balanced guidance [4]. Concerns have also been raised regarding weight management during pregnancy. Many women express disappointment with the advice provided by midwives, particularly in relation to gestational weight gain (GWG) [5]. In some cases, weight is rarely mentioned, leading women to believe that GWG is unimportant, even when they have personal concerns about their weight. Midwives themselves have reported feeling uncomfortable discussing weight, often avoiding the topic due to fears of causing offence [6].

Despite the importance of nutrition, weight management, and physical activity during pregnancy, midwives often receive limited training on these topics. This education gap is evident both during pre‐registration training and through Continuous Professional Development (CPD) opportunities [6, 7, 8]. As a result, midwives may feel underprepared to deliver comprehensive guidance in these areas, which can impact the quality and consistency of nutritional support provided to pregnant women.

To ensure that key, evidence‐based messages are communicated effectively during antenatal care, greater investment is needed to enhance midwives' knowledge and confidence in providing guidance on nutrition and GWG. However, before implementing training interventions, it is essential to engage with midwives and others to ensure that any resources or educational initiatives are relevant, practical and aligned with their needs. Stakeholder engagement plays a crucial role in shaping effective interventions, fostering collaboration, and ensuring that those directly involved in antenatal care can contribute their perspectives to the development process [9]. When such work involves patients, it is called ‘Patient and Public Involvement’ (PPI) [10]. By prioritising such engagement, future initiatives can be better tailored to support midwives in delivering high‐quality nutritional care, ultimately improving pregnancy outcomes for mothers and their babies.

### 1.1 Aim

To engage antenatal midwives in stakeholder discussions to explore their perspectives on the design and delivery of a nutrition resource tailored to their needs.

## 2 Methodology

This study employed a qualitative research design underpinned by a constructive ontology and a qualitative, interpretive epistemology. A constructivist stance acknowledges that reality is socially constructed, shaped by individual experiences and interactions. From an interpretivist perspective, knowledge is co‐created between researchers and participants, recognising the subjective nature of meaning‐making in qualitative enquiry. In line with these philosophical assumptions, the study adopted a Participatory Action Research (PAR) methodology [11], actively involving midwives in shaping discussions and co‐producing solutions related to diet and weight management in antenatal care. PAR, as described by Baum, MacDougall, and Smith [11], is grounded in principles of collaboration, reflexivity, and action‐oriented knowledge generation, positioning participants as co‐researchers in identifying and addressing practice‐based challenges.

Reflexive Thematic Analysis [12, 13] was chosen as the analytic approach, as it allows for a nuanced exploration of participants' experiences, acknowledging the active role of the researcher in interpreting data. This method aligns with the study's aim of engaging midwives in discussions about a nutrition resource, ensuring that their perspectives are central to the knowledge generation process. Ethical consultation was conducted with the Edge Hill University (Health) Research Ethics Committee, which confirmed the study met criteria for exemption as Stakeholder, Patient and Public Involvement (PPI) activity. We sought informed consent from participants to share their views in future research design and through research outputs.

### 2.1 Recruitment and Participants

The lead researcher approached the midwifery managers at a local NHS hospital that provided antenatal care, both within the hospital and through community clinics. The research was conducted in a large urban NHS maternity service in Northwest England. Community midwives operated across multiple Children's Centres serving deprived, multicultural populations, while hospital midwives worked in a busy, consultant‐led antenatal clinic. Community and hospital‐based midwives had overlapping but distinct roles, with community staff typically providing continuity of care, including home visits, and hospital midwives delivering more complex, high‐risk antenatal care. After obtaining permission, the researcher attended routine midwifery meetings to introduce the study and to collect feedback. These meetings provided an opportunity to invite midwives in antenatal care to participate in research workshops, where they could share their thoughts and expertise on the proposed resource.

A total of 26 midwives participated in four workshops; two were conducted at Children's Centres (W1, *n* = 9 and W2, *n* = 10), representing 19 community midwives, and two at antenatal clinics within local hospitals (W3, *n* = 3 and W4, *n* = 4), representing seven hospital‐based midwives. Each workshop ran for approximately an hour (giving a total of 4 h of workshop discussions).

### 2.2 Participatory Action Research Workshop Content and Data Collection

The workshops were designed to foster engagement, encourage shared learning, and ensure that participants had an integral role in articulating both the barriers they face and the practical solutions they envisage. A semi‐structured workshop guide was developed by the research team, informed by insights from prior Patient and Public Involvement and Engagement (PPIE) activities, as well as previously published qualitative research involving midwives and women who were pregnant or recently postpartum [4, 5, 6]. Each workshop began with a brief introductory presentation by the lead researcher to set the context. This included an overview of previous research highlighting knowledge gaps in midwives' advice on diet and weight, followed by an explanation of the workshop's purpose and the intended focus of the discussions. The workshop was then purposefully structured in two interlinked phases to facilitate a reflective and co‐productive process.

Phase 1: Surfacing Practice‐Based Realities

The phase centred on an open, inductively driven discussion of midwives' lived experiences delivering antenatal diet, nutrition and weight. Rather than following a predefined framework, this phase allowed participants to freely explore and articulate the challenges, gaps, and contextual realities they encountered in practice. This grounding phase was critical in surfacing authentic, experience‐led concerns and priorities from the midwives themselves. To support structured yet open dialogue, participants engaged in a visual mapping exercise designed to facilitate group discussion and facilitate collaborative meaning‐making. Midwives were initially encouraged to share statements and notes based on their recent experiences of discussing nutrition and weight with pregnant women. These reflections were captured in real time, individually or collaboratively, using post‐it notes and marker pens to promote inclusivity, visibility of ideas and ease of contribution.

**Figure 1:**
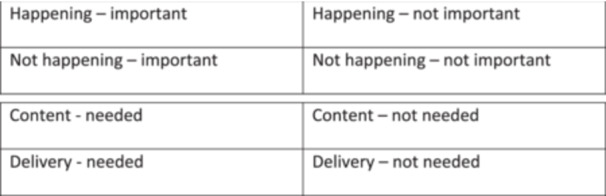
Matrix one and two.

Subsequently, participants were asked to organise their contributions onto a four‐quadrant matrix, evaluating each item in terms of its perceived importance and whether it was currently enacted in practice. The quadrants included [1]: important and happening [2]; important but not happening [3]; not important but happening; and [4] not important and not happening (Figure 1). This interactive exercise enabled participants to visualise the alignment, or disconnect, between priorities and existing service delivery. The categorisation process prompted more nuanced dialogue, as participants collectively interrogated each issue's significance, feasibility, and systemic conditions. It also fostered stronger group dynamics and ensured broad participation across the workshop. The act of translating their reflections into a shared visual artefact supported the co‐construction of meaning but also served as a catalyst for the next stage of the workshop, which focused on solution generation.

Phase 2: Co‐producing Resource Solutions

This phase focused on the co‐development of a practical resource to support midwives in addressing the identified gaps. This phase adopted a solution‐focused lens; enabling participants to transition from identifying challenges to collaboratively generating meaningful responses grounded in their professional experiences. A second visual mapping activity structured this discussion. Participants were asked to contribute ideas related to the potential content and delivery of a midwifery nutrition resource, categorised across four domains [1]: content needed [2], content not needed [3], delivery methods needed, and [4] delivery methods not needed (Figure 1). Post‐it notes and marker pens were again used to encourage individual reflection and collective discussion.

This exercise encouraged participants to critically appraise existing practices while envisioning improvements grounded in feasibility and acceptability. Participants shared preferences related to the modality of training (e.g., face‐to‐face vs e‐learning), the design of printed and visual materials, and the extent to which the resource could accommodate diverse populations and service settings. There was a shared emphasis on ensuring the resource was inclusive, practical, and adaptable, reflective of midwives' capacity and the socio‐cultural needs of pregnant women.

To consolidate and expand this co‐design process, participants were invited to consider the broader question: “*What does best practice look like*?” in relation to antenatal diet and weight management advice. These insights were captured on a third flip chart sheet, surfacing aspirational yet actionable examples of effective communication, resource use and care integration.

The workshops were facilitated by the lead researcher, who employed open‐ended prompts and active listening to support inclusive participation and deepen reflection. Detailed field notes were recorded following each session to contextualise the visual data, document group dynamics, and capture subtler dimensions of meaning not immediately evident in written materials. This participatory and iterative process ensured that midwives were not positioned as passive informants, but as co‐producers of knowledge and practice‐enhancing solutions. IT aligned with the emancipatory ethos of PAR by valuing professional expertise, enabling reflectivity and generating outputs directly relevant to midwifery care.

### 2.3 Data Analysis

Data were analysed using Reflexive Thematic Analysis (RTA), as outlined by Braun and Clarke [12, 13], to explore midwives' perspectives and co‐develop a nutrition resource relevant to antenatal care. An inductive, data‐driven approach was adopted to ensure that the themes generated were grounded in participants' lived experiences and co‐constructed meaning‐making.

The analysis process followed six recursive phases (Table 1):

**Table 1:** Thematic analysis process (informed by Braun and Clarke [12, 13]).

Familiarisation	Participants' written notes, flipchart content, photographs, post‐it note contributions, verbatim quotations, and detailed researcher field notes, including verbatim quotes from participants at each workshop, were reviewed repeatedly by the lead researcher to immerse themselves in the data. Initial insights and reflexive notes were documented during this phase to begin identifying patterns of meaning.
Generating Initial Codes	All textual data, including verbatim workshop comments and visual materials, were coded using an open, semantic coding approach. Coding was conducted iteratively to capture the diversity of views across hospital‐ and community‐based midwives. Codes reflected both explicit content and contextual nuance, acknowledging the interactive and participatory setting of the data collection.
Constructing Initial Themes	Codes were reviewed and clustered into potential themes by examining shared meanings and conceptual connections. Regular discussions within the multidisciplinary research team supported the interpretation of codes and refinement of theme boundaries.
Reviewing Themes	The preliminary themes were reviewed and refined to ensure internal coherence and meaningful distinction. Data extracts were re‐examined to confirm their alignment with the developing thematic structure.
Defining and Naming Themes	Final themes were developed to provide a coherent narrative reflecting the data. Each theme was clearly defined to articulate its scope, analytical focus, and relevance to the study's aim. Reflexivity was maintained throughout to critically appraise the researchers' interpretive influence on theme construction.
Producing the Report	The final themes were synthesised into four overarching thematic areas, integrating representative data extracts and interpretive commentary. The findings were presented to a subset of participants by the PPIE representative to enhance validity through member reflection. An audit trail of decisions, coding matrices, and thematic maps was maintained to support transparency and rigour.

This analysis process ensured that stakeholder voices were central to knowledge generation, aligning with the principles of participatory action research and enhancing the utility of findings for midwifery practice and resource development.

### 2.4 Research Team

This study was conducted by a multidisciplinary research team with expertise spanning maternal nutrition, health psychology, obesity, healthcare service development, and qualitative research methodology. The lead researcher, a Registered Dietitian and Reader in Dietetics, has a specialist research interest in maternal nutrition and led the study design and data collection. A Health Psychologist and Reader in Applied Health Psychology, with expertise in obesity and health professional service development, contributed to the study's theoretical framework and qualitative research methodology. To incorporate INVOLVE [10] and GRIPP2 [14] guidelines in the development of this research, a Midwife (H.R.) and two PPI representatives from multicultural backgrounds (C.B., E.R.) were recruited to ensure that the study remained relevant and meaningful to both midwives and service users. They were briefed regarding the purpose of the role and all contributed to analytical discussions, edits of this article and are named co‐authors receiving a fee in line with INVOLVE guidelines [10]. The interdisciplinary nature of the research team strengthened the study's design, data interpretation, and the applicability of findings to practice.

### 2.5 Ensuring Quality in Qualitative Research

Several strategies were implemented throughout the research process to ensure the quality of the qualitative research conducted. Immediately after each workshop, the lead researcher (J.A.) engaged in reflexive discussions and note‐taking with another member of the research team (L.N.). During analysis, reflexivity was maintained by the research team through ongoing discussions, acknowledging how our professional backgrounds and perspectives may influence data collection and interpretation. Triangulation was achieved through the diverse expertise of the research team, allowing for multiple perspectives to be considered in the analysis. The study adhered to Braun and Clarke's [12, 13] approach to Reflexive Thematic Analysis, ensuring a systematic and transparent coding process. Additionally, an audit trail was maintained, documenting key decisions throughout data analysis to support the transparency and trustworthiness of findings. These measures collectively ensured that the study's findings were robust, reflective of participant perspectives, and contributed meaningful insights to antenatal midwifery practice.

## 3 Results

**Figure 2:**
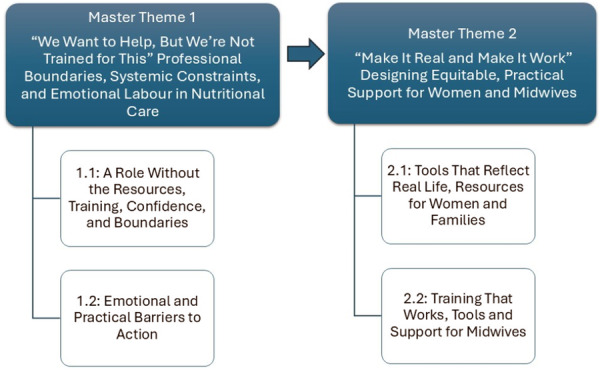
Thematic Map.

The stakeholder engagement workshops provided rich, nuanced insights into midwives' experiences of delivering nutritional advice in antenatal care. Through inductive and interpretative analysis, two master themes (Figure 2) were constructed to capture midwives' reflections on how they made sense of their roles, constraints, and aspirations in providing nutritional advice. These themes reflect both the felt challenges of practice and the imagined possibilities for change, grounded in the real‐world complexity of antenatal care. The first master theme, “We Want to Help, But We're Not Trained for This”: Professional Boundaries, Systemic Constraints, and Emotional Labour in Nutritional Care”, is explored through two subthemes. The second master theme, “Make It Real and Make It Work”, Designing Equitable, Practical Support for Women and Midwives” is examined through two interconnected subthemes. Each theme is discussed below and supported by illustrative quotations.

### 3.1 Master Theme 1: “We Want to Help, but We're Not Trained for This”: Professional Boundaries, Systemic Constraints, and Emotional Labour in Nutritional Care

Midwives consistently expressed a strong motivation to support women with tailored nutritional advice during antenatal care. However, they described a pervasive tension between professional aspiration and the realities of practice. This theme explores how limited training, ambiguous role boundaries, structural barriers, and the emotional complexity of nutritional conversations constrained midwives' capacity to deliver meaningful support. Two interrelated subthemes illustrate the practical and emotional consequences of this disconnect.

#### 3.1.1 Subtheme 1.1: A Role Without the Resources, Training, Confidence, and Boundaries

Across all workshops, midwives reflected on their limited education and training in antenatal nutrition. Despite being expected to deliver guidance on diet, weight, supplementation, and physical activity, many felt ill‐equipped to do so confidently or accurately. Most reported that their nutritional knowledge was based on personal experience rather than formal education, and they had received little or no targeted CPD to develop this aspect of their role. They highlighted their lack of confidence in providing advice tailored to specific dietary needs, particularly for women who were vegetarian, vegan, or followed religious or culturally specific diets. Without clear frameworks or training on diverse nutritional practices, they feared giving incorrect or culturally insensitive information.

Different women need different advice.

(W2: Community midwife)

While signposting to NHS resources or referring to dietitians was a common strategy, midwives often described this as a compromise due to their own limitations. An illustrative example was the rising prevalence of iron‐deficiency anaemia. Several midwives viewed this as an expensive, largely preventable issue that could be addressed with earlier nutritional intervention.

We are seeing so much anaemia… most of this could be prevented with the right conversations early on.

(W4: Hospital midwife)

They felt the expansion of their responsibilities around public health, without adequate investment in knowledge or resources, had led to blurred role boundaries and professional unease. They described a widening gap between what is clinically needed, what policy expects, and what they feel prepared or permitted to deliver in practice.

#### 3.1.2 Subtheme 1.2: Emotional and Practical Barriers to Action

Beyond skills and knowledge, midwives also described the emotional toll of engaging in sensitive nutritional discussions. Topics such as weight, eating habits, and food insecurity were often perceived as emotionally charged, requiring delicacy, empathy, and significant relational skills.

When you're asking such personal questions, women often feel judged… You need to be so careful how you phrase things.

(W2: Community midwife)

Time pressures were the most frequently cited barrier to delivering effective care. Midwives reported that antenatal appointments were already overstretched, with competing priorities leaving little room for complex or relational conversations about food and health behaviours.

We just don't have the time to get into it properly, it's not that we don't care, it's that we're already stretched.

(W1: Community midwife)

Midwives expressed frustration with nutrition resources currently available; they were described as too generic, offering little help in facilitating brief, person‐centred interventions during routine appointments. The limitations of using Body Mass Index during pregnancy were emphasized. They expressed a strong need for training in communication skills, allowing them to deliver non‐judgemental advice. Training that focused not only on nutritional content but also on how to talk about it, with compassion and confidence, was essential for making any guidance deliverable within real‐world practice.

#### 3.1.3 Summary

While midwives saw themselves as well‐placed to initiate these conversations, they felt professionally, emotionally, and practically underprepared to do so. Their reflections point to an urgent need for context‐sensitive training, clearer role guidance, and emotionally intelligent tools that align with the lived realities of antenatal practice.

### 3.2 Master Theme 2: “Make It Real and Make It Work” – Designing Equitable, Practical Support for Women and Midwives

Midwives in this study offered a co‐produced vision for how antenatal nutritional care could be improved to better reflect the realities of practice and the needs of diverse populations. Their reflections centred on two interconnected priorities: (1) the urgent need for practical, inclusive resources that resonate with women's everyday lives; (2) the development of usable tools and training to support midwives in delivering effective, culturally sensitive advice. This overarching theme is organised into two subthemes that illustrate how midwives envisioned more equitable, realistic, and sustainable models of antenatal nutritional support.

#### 3.2.1 Subtheme 2.1: Tools That Reflect Real Life, Resources for Women and Families

Midwives described a disconnection between current nutrition resources and the socioeconomic realities of the women they support. Many noted that national guidance and materials tend to assume a level of financial security, digital access, and cultural familiarity that is far from universal. The over‐reliance on online platforms was viewed as exclusionary, particularly for women experiencing material deprivation or limited digital literacy.

Not everyone has access to a computer or a smartphone, it's not realistic to assume all women can look things up online.

(W1 – Community midwife)

Participants expressed a preference for printed visual resources that could be physically shared in clinic settings. The removal of trusted tools such as ‘The Pregnancy Book’ was viewed as a significant loss, not only in terms of accessibility but also as a consistent and inclusive source of information.

We used to hand out The Pregnancy Book…now we refer them to a website.

(W4 – Hospital midwife)

Midwives called for new resources that were multilingual, culturally appropriate, low‐literacy, and grounded in the realities of women's day‐to‐day lives. They described the need for tools that reflect diverse cooking practices, budgeting constraints, and family structures, including culturally familiar foods and shopping strategies relevant to different communities.

It's hard to talk about healthy eating when people cannot afford to eat.

(W1: Community midwife)

Persistent myths and misinformation around nutrition were a further challenge. Midwives felt poorly equipped to challenge these without credible materials that could be used within brief appointments.

There is so much information online…it's hard to know what to believe.

(W1 – Community midwife)

Resources were not only described as educational tools, but as relational ones: a means of building trust, engaging families, and facilitating open, non‐judgemental conversations. Participants emphasised the need to move beyond a woman‐focused model and consider nutrition in the wider household context, particularly given the influence of partners, elders, and family traditions on dietary behaviours.

Advice should be for the whole family, not just women. They're not just feeding themselves.

(W2 – Community Midwife)

#### 3.2.2 Subtheme 2.2: Training That Works, Tools and Support for Midwives

Midwives consistently described gaps in their own training and support. They expressed a desire for practical, in‐person training led by experts, particularly dietitians, who could provide not only content expertise but real‐time engagement through case examples, discussion, and role‐play.

Delivered by someone who knows what they are talking about…like you! (Specialist dietitian).

(W3 – Hospital Midwife)

They proposed a ‘filofax‐style’ portable resource, including concise prompts, myth‐busting fact sheets, and visual aids to support culturally tailored and brief interventions. Such tools were preferred over digital platforms, which were often inaccessible in clinic settings.

A quick‐glance guide, something you could just flip through with a woman in clinic, that would be amazing.

(W1 – Community midwife)

Participants aligned their vision with the Making Every Contact Count (MECC) approach [15]; brief interventions embedded into routine care, but emphasised that to make this meaningful, they needed tools that worked within time constraints and could be adapted to different service contexts. Structural inequities in service provision were also raised. Midwives described regional disparities in access to specialist support such as dietitians, social prescribers, interpreters, and parenting education services.

Classes should reach the right people…those who are most in need.

(W2 – Community Midwife)

They advocated for greater integration between maternity services and wider systems (e.g. the Healthy Child Programme, Healthy Start), and for consistent investment in equitable referral pathways across the care continuum.

#### 3.2.3 Summary

This theme brings together midwives' insights into how nutrition care can be made more equitable, usable, and embedded in everyday antenatal practice. Their vision reflects a deeply relational and context‐sensitive understanding of women's and practitioners' needs. Midwives recognised that supporting nutritional well‐being requires more than disseminating advice; it requires tools that are culturally resonant, accessible, and designed with both clinical workflow and family dynamics in mind. Importantly, this theme highlights the interdependence of midwives' training and women's empowerment. Without tools that enable midwives to act with confidence and resources that meet women where they are, national guidelines risk being aspirational but ultimately unimplemented. These findings support a shift towards co‐designed, community‐informed, and system‐integrated models of care that bridge the divide between policy and practice.

## 4 Discussion

This appears to be the first paper to seek the views and opinions of midwives regarding the design and co‐creation of a nutrition resource to support their conversations about healthy eating during antenatal care. Midwives consistently highlighted a desire to provide personalised, meaningful advice to women about diet, weight, and nutritional well‐being. Yet, their accounts revealed a systemic misalignment between national policy expectations and local, day‐to‐day practice realities. They reported limited training, insufficient confidence, and a lack of appropriate tools to deliver nuanced advice, especially for women with cultural, religious, or economic needs. This reflects broader health inequalities and the influence of social determinants of health, particularly for women living in areas of deprivation, from ethnic minority backgrounds, or facing food insecurity. It also echoes wider evidence that midwives often operate at the limits of their professional competence in areas where interdisciplinary support is lacking [6].

While the recently published Maternal and Child Nutrition guidelines [2] represent a welcome advancement in the scope and specificity of antenatal nutrition recommendations, this study highlights a persistent gap between clinical guidance and practical implementation. Although the updated guidelines provide detailed advice on what topics to cover, such as healthy eating, supplement use, and weight management, they remain silent on the how, who, when, and where of delivery within real‐world maternity care. Midwives expressed uncertainty about their remit in providing nutritional advice, but also about how to translate generic guidance into contextually appropriate, culturally responsive conversations. Structural barriers, such as time constraints, limited training, and the absence of practical tools, further constrain the effective uptake of national policy. These issues are compounded by postcode‐driven variations in service access and resource availability, contributing to health inequities that undermine the goals of the Healthy Child Programme [16], which seeks to provide universal and proportionate care in early life. As such, the presence of more detailed guidelines does not address the mechanisms through which care can be operationalised equitably or sustainably. This reinforces a growing body of evidence across behavioural medicine and implementation science [17, 18] that policy‐level guidance must be supported by capacity‐building, interprofessional collaboration, and system‐level public health changes to realise impact in maternal and child health [19].

Professionally, midwives expressed a tension between their holistic care ethos and perceived role boundaries. Many described themselves as ‘sign posters’ rather than advisors, yet demonstrated clear insight into preventable nutritional harms such as anaemia and gestational diabetes mellitus. They also referred to the role of Health Visitors, who also have a responsibility for health promotion in the antenatal and postnatal periods. This reveals both an unmet need and untapped potential: midwives want to do more but lack the system‐level scaffolding, through training, time, or pathways, to do so effectively. These findings parallel previous studies on midwifery identity, relational care, and the importance of interdisciplinary support [20]. Without such systemic investment, aspirations to reduce preventable maternal and child health outcomes through upstream interventions will remain unfulfilled. From a dietetics perspective, the study highlights the persistent gap between population‐level nutrition guidelines and frontline antenatal implementation. Participants advocated for flexible, practical resources, such as visual toolkits, low‐literacy materials, and culturally responsive fact sheets that reflect the lived experience of pregnant women, especially those experiencing food insecurity or socioeconomic disadvantage. Concerns about iron deficiency and the rising use of costly intravenous iron infusions, viewed as largely preventable, highlight the implications for NHS resource use and health service planning.

These co‐produced ideas align with translational research in public health nutrition and implementation science, advocating for tools that are not only evidence‐based but also socially and contextually meaningful [21]. Addressing food system issues, such as the affordability, accessibility, and cultural relevance of healthy food, must become a core concern of antenatal health promotion efforts. Midwives stressed the importance of a holistic approach, not just focusing on pregnant women but considering the whole family's nutrition. They emphasised that any advice given must be realistic, practical, and tailored to fit women's daily lives, particularly for those experiencing financial hardship or limited food security. Many women, especially those in deprived areas, struggled to afford nutritious food, making it even more critical to provide structured referrals for practical support, such as budgeting, cooking skills, and meal planning, potentially through social prescribing services. Maternity Action highlighted the impact of the UK's current cost‐of‐living crisis on pregnant women in their latest survey [22]. Half of the 1176 respondents reported buying less healthy food due to increased costs, with 38% eating less or skipping meals. One in four (24%) reported going without food during their pregnancy, to feed their children [22]. The discussions with midwives revealed that they were very aware of the hardships that many of their women were facing and reported that they frequently refer to Food Banks and Food Pantries for support. Maternity Action, has called for urgent investment in increasing maternity pay in line with inflation and an end to treating Maternity Allowance as earned income within Universal Credit, as this unfairly penalises pregnant women and their families [22]. Schemes such as ‘Healthy Start’, which provide pregnant women in receipt of certain benefits (in England) with additional funds to buy milk, fruit and vegetables during pregnancy, have not kept abreast with inflation either. Currently, recipients are entitled to £4.25 per week from 10 weeks' gestation [23].

Likewise, midwives expressed frustration that nutritional advice often arrives too late in the care pathway, missing the critical first trimester and pre‐conception period. In contrast to the UK, countries like the United States offer detailed preconception care guidelines encompassing nutrition, physical activity, and weight management [24]. This gap emphasises the need to extend antenatal nutrition strategies into earlier stages of the reproductive life course and to integrate consistent messages about healthy eating during pregnancy into the public health initiatives across communities.

Midwives thought that conversations around diet and weight should be normalised rather than stigmatised, allowing for open, non‐judgmental discussions that address common misconceptions. Earlier research has highlighted that midwives often avoid talking about weight, due to a lack of clear guidance regarding optimal gestational weight gain, but also because they worry about offending people [6]. Furthermore, Charnley et al., [25] reported that pregnant women living with obesity could recount unpleasant and judgemental comments made by healthcare staff during their antenatal care. It is well documented that people who experience weight stigma are less likely to engage with healthcare services and healthy behaviour; stigma may also trigger over consumption of food or binge eating [26]. So, midwives are right to be cautious when initiating conversations about weight. In addition to concerns around stigma and health behaviours, it is important to recognise that many women living with obesity may also experience disordered eating or complex relationships with food. Restrictive eating, binge eating, or emotional eating are frequently under‐recognised in antenatal care, yet may coexist with nutritional deficiencies, especially in women with higher BMIs. Research by Charnley et al., [25] highlights that women living with obesity during pregnancy can simultaneously face nutritional insufficiency, such as iron deficiency or low vitamin D levels, despite presenting with overweight or obesity. This paradox highlights the need for midwives to be supported with education that goes beyond weight‐based guidance and encompasses the psychological, behavioural, and nutritional dimensions of maternal health. Embedding this into antenatal education and training would help midwives better identify risk, respond sensitively, and provide support that is psychologically informed and nutritionally sound. The midwives also highlighted the need to recognise the psychological dimensions of food and eating, particularly the impact of mood, cravings, and pregnancy‐related changes in appetite. Including the expertise of a health psychologist in the development and delivery of the nutrition resource may help to build the confidence and skills of midwives regarding sensitive but effective discussions about weight.

Time pressures and emotional labour were persistent barriers. Midwives described the difficulty of navigating conversations that may be perceived as judgmental or stigmatising, particularly in relation to weight or food insecurity. Their calls for communication skills training, grounded in empathy, cultural humility, and behavioural science, resonate with health psychology literature on motivational interviewing, stigma avoidance, and self‐determination theory [27, 28]. Addressing these challenges is critical to mitigate stigma and prevent disengagement from antenatal services, especially among women at the intersection of multiple disadvantages. A key aspect of best practice identified by midwives, involved the strengthening of referral pathways, ensuring they could easily connect women to specialist dietetic advice and structured weight management services where necessary. National Institute for Health and Care Excellence (NICE) [2] currently recommends that anyone booking in for antenatal care with a BMI > 40 kg/m^2^ should be offered referral for specialist advice and support, yet midwives in this study did not appear to be confident in initiating such referrals, suggesting that there were no clear pathways during pregnancy. Midwives' recommendations for interactive, dietitian‐led training and ‘filofax‐style’ tools reflect a practical and co‐constructed vision for best practice. Their ideas align closely with MECC, a public health approach advocating for brief but meaningful health conversations during routine care [15]. NICE suggests that staff in regular contact with people could provide them with a brief intervention, typically taking no more than a few minutes and involving a discussion about making simple changes to improve their lifestyle [29]. Such an approach would not necessarily add to the workload of busy healthcare professionals, such as midwives. This is consistent with recommendations from the Royal College of Obstetricians and Gynaecologists (RCOG), which emphasise the importance of brief, sensitive, and non‐stigmatising conversations around weight, physical activity, and nutrition in antenatal care. The RCOG Green‐top Guideline [30] provides comprehensive guidance for the care of women with obesity during pregnancy, advocating for early intervention, structured pathways, and appropriately trained staff to support maternal and foetal outcomes. However, our findings suggest that midwives often lack the time, resources, or confidence to deliver these interventions effectively without additional support or training. This highlights the need for system‐level changes to operationalise these guidelines in practice.

The study also speaks to the importance of systems‐level change. Digital inequalities, lack of printed materials, and postcode‐driven variation in access to specialist support were seen as major contributors to health inequities. Midwives argued for more equitable models of antenatal nutrition care, including structured referral pathways to dietitians, social prescribers, and parent education programmes. Theoretical frameworks from behavioural medicine, including socioecological models and the Behaviour Change Wheel [28, 31], reinforce the need for multi‐level interventions that span individual, organisational, and policy domains. Midwives' recognition of the wider household context of food practices adds further depth. Nutrition was framed not simply as an individual health behaviour but as embedded within families, social roles, and cultural traditions. Their suggestion to expand interventions to partners and family members reflects evidence on the collective influences on maternal nutrition and health behaviours [32]. This relational perspective calls for more family‐inclusive and community‐based models of antenatal care, central to the Healthy Child Programme's vision of whole‐family support from preconception through early years.

### 4.1 Implications for Public Health and Maternity Policy and Practice

This PPI study offers critical insight for public health systems aiming to advance maternal nutrition and reduce perinatal health disparities. It highlights the need to integrate nutrition and food security more coherently into public health and maternal health systems, ensuring upstream support that begins at pre‐conception and spans the family unit. Policies must recognise the social determinants that shape dietary behaviours. Closing the implementation gap requires not only clinical guidelines but also a supportive public health infrastructure that empowers midwives, through training, time, and tools, to act as agents of health equity. System‐wide approaches that embed co‐production, interprofessional collaboration, and social prescribing within routine maternity care are essential. In doing so, public health policy can move beyond aspirational statements to deliver on its commitment to reduce health inequalities, transform food systems, and support every child's right to a healthy start. The 2010 Marmot Review [33] called for effective strategies for reducing health inequalities, notably advocating for giving every child the best start in life. However, despite these recommendations, health inequalities in England have worsened. In 2020, Marmot [34] emphasised the need for a renewed focus on early years, including antenatal care, to address these disparities. The midwives in this study identified gaps in pre‐conception and early pregnancy support, recognising that women often do not receive nutritional guidance until after 12 weeks of pregnancy, missing the critical early period for foetal development. We argue that systematically integrating nutritional guidance early in pregnancy, alongside extending the Healthy Child Programme's 28‐week check, could provide more targeted nutritional advice to support maternal and child health outcomes. In 2024, the House of Lords Food, Diet and Obesity Committee released a report [35] reinforcing the importance of early nutrition, recommending the development of a Maternal and Infant Nutrition Strategy. A key aspect of this strategy should be the inclusion of maternal and antenatal nutrition advice, with midwives working alongside health visitors to support a more cohesive approach to maternal care.

### 4.2 Strengths and Limitations

A key strength of this study is its applied PAR approach, which underpinned both the workshop design and subsequent analysis. By embedding co‐design methodology into the structure of the workshop, the study enabled midwives to actively reflect on practice and contribute to shaping future resource development. This approach not only enhanced the ecological validity of the findings but also ensured that the themes and recommendations were grounded in practitioner‐led insight and situated knowledge. We consider our analytical reflection a strength of this study. This thematic structure reflects a reflexive engagement with midwives' narratives, foregrounding their agency while situating their experiences within broader systemic, professional, and interpersonal contexts. Guided by Braun and Clarke's [13] reflexive thematic analysis, our analysis moves beyond surface‐level description to interpretive insights about the relational and structural dynamics shaping antenatal nutritional care. Themes were developed inductively and iteratively, shaped by the research team's critical reflection on both the content and context of the data. An additional strength of this study lies in the involvement of a multidisciplinary research team, including expertise in dietetics, health psychology, public health, and midwifery, with direct experience working in or alongside NHS maternity services. This shared familiarity with clinical practice, operational constraints, and team dynamics enriched the delivery of the workshops to aid engagement and discussion and, in addition, enriched the analysis by grounding it in the realities of antenatal care delivery. The presence of a practising midwife on the research team added depth and contextual sensitivity, particularly in attending to professional identity, the emotional labour of care, and how structural constraints manifest in everyday practice. This insider perspective was critically balanced within a broader interpretive dialogue, enabling closeness to and distance from the data. The analytic process was further enhanced through triangulation, involving two recently postpartum women, supporting a more inclusive, socially situated understanding of the data. All participant midwives were women. While this aligns with the demographics of the UK midwifery workforce, it may have influenced perspectives on communication, empathy, and engagement in nutritional discussions, particularly regarding culturally sensitive or gendered dynamics. Midwives were recruited from both hospital and community settings, with varying years of experience, allowing a diversity of experiences and reflections across service contexts and supporting the transferability of findings to other maternity care services. However, it is noteworthy that the research was conducted in a single urban area in Northwest England, and while rich in context, the findings may not fully represent experiences in other regions or rural settings. Antenatal care pathways vary across the UK and internationally, and thus, the transferability of findings may be constrained by local organisational structures and service models. Health visitors also have a role, providing public health antenatal and postnatal advice to families, but were not included in this study. Nevertheless, the integration of clinical, academic, and experiential perspectives, combined with reflexive and inclusive analytic practices, supports the trustworthiness and relevance of the findings. The co‐production of themes with attention to lived experience and professional practice positions this study as a robust contribution to the ongoing conversation about improving nutritional care in maternity services.

## 5 Conclusion

Midwives expressed a strong need for improved nutrition training and resources, highlighting their current lack of confidence in providing dietary advice. Financial barriers, inconsistent access to information, and limited consultation time were seen as key challenges. Despite acknowledging their limited time and inability to change healthcare systems, their recommendations focused on practical, evidence‐based interventions that empower both midwives and pregnant women to make informed decisions about their nutrition. By integrating training, resources, and support systems, midwifery‐led nutritional care can be enhanced, ultimately improving maternal and infant health outcomes.

## Author Contributions

**Julie Abayomi:** conceptualization, investigation, funding acquisition, writing – original draft, methodology, validation, visualization, writing – review and editing, formal analysis, project administration. **Helen Richards:** assisted with analysis and interpretation of the findings, reviewed and approved the final paper. **Camila Benavides:** assisted with analysis and interpretation of the findings, reviewed and approved the final paper. **Ekaterina Reyneke:** assisted with analysis and interpretation of the findings, reviewed and approved the final paper. **Lisa Newson:** writing – original draft, conceptualization, formal analysis, methodology, writing – review and editing.

## Conflicts of Interest

The authors declare no conflicts of interest.

## Data Availability

The authors have nothing to report.
